# Versorgungsrealität des Ösophaguskarzinoms im Bundesland Brandenburg: Ein Vergleich zwischen Plattenepithel- und Adenokarzinomen

**DOI:** 10.1007/s00104-024-02110-0

**Published:** 2024-06-11

**Authors:** Andreas Loew, Constanze Schneider, Maren Pflüger, René Mantke, Karsten H. Weylandt, Stephan Gretschel

**Affiliations:** 1 Medizinische Klinik B, Universitätsklinikum Ruppin-Brandenburg (ukrb), Medizinische Hochschule Brandenburg, 16816 Neuruppin, Deutschland; 2 Klinisch-epidemiologisches Krebsregister Brandenburg-Berlin (KKRBB), 03044 Cottbus, Deutschland; 3 Klinik für Allgemein- und Viszeralchirurgie, Universitätsklinikum Brandenburg, Medizinische Hochschule Brandenburg, 14770 Brandenburg an der Havel, Deutschland; 4 Fakultät für Gesundheitswissenschaften, gemeinsame Fakultät der Brandenburgischen Technischen Universität Cottbus-Senftenberg, der Brandenburgischen Medizinischen Hochschule Theodor Fontane und der Universität Potsdam, Potsdam, Deutschland; 5 Klinik für Allgemein‑, Viszeral‑, Thorax- und Gefäßchirurgie, Universitätsklinikum Ruppin-Brandenburg (ukrb), Medizinische Hochschule Brandenburg, 16816 Neuruppin, Deutschland

**Keywords:** Ösophagus, Plattenepithelkarzinom, Adenokarzinom, Epidemiologie, Therapie, Oesophagus, Squamous cell carcinoma, Adenocarcinoma, Epidemiology, Therapy

## Abstract

**Hintergrund:**

Klinische Krebsregister sollen durch differenzierte Datenauswertung die Versorgungsrealität abbilden und ggf. Ansätze für Verbesserung der Versorgung bieten.

**Methode:**

Für die Jahre 2000 bis 2018 wurden die Daten des klinisch-epidemiologischen Krebsregisters Brandenburg-Berlin bezüglich Epidemiologie und Versorgungsrealität getrennt nach Adeno- und Plattenepithelkarzinom untersucht.

**Ergebnisse:**

Zwischen 2000 und 2018 wurden 3207 Ösophaguskarzinome im Krebsregister dokumentiert, davon waren 2182 Plattenepithelkarzinome (ESCC), 843 Adenokarzinome (EAC) und 182 verschiedene andere oder fehlende Histologien. Im Beobachtungszeitraum zeigte sich eine deutliche Dominanz der ESCC, jedoch mit einer signifikanten Zunahme der EAC bei beiden Geschlechtern. Die Neuerkrankungsrate war insgesamt für Männer 5fach höher als für Frauen. Die relative 5‑Jahres-Überlebenswahrscheinlichkeit aller Ösophaguskarzinome lag bei Männern bei 17,4 % und bei Frauen bei 22,5 %. Patienten mit EAC überlebten signifikant länger als mit ESCC. Als Therapiemethoden kamen überwiegend Strahlen- und Chemotherapie, einzeln oder in Kombination, zum Einsatz. Operiert wurden 19 % der Plattenepithel- und 42 % der Adenokarzinome.

**Schlussfolgerung:**

Der Anteil der ESCC ist in Brandenburg immer noch deutlich höher als der der EAC, wobei für Letztere, insbesondere bei Männern, ein signifikanter Anstieg zu verzeichnen ist. Obwohl lokal fortgeschrittene Tumoren deutlich häufiger auftraten, sind moderne neoadjuvante Konzepte bisher selten dokumentiert und obwohl die Qualität der Operationen mit dem internationalen Standard vergleichbar ist, werden nur relativ wenige Patienten operiert.

## Abstract (en)

**Background:**

Clinical cancer registries are intended to reflect the reality of care through differentiated data analysis and, if necessary, to offer approaches for improving care.

**Methods:**

For the years 2000–2018, the data of the Clinical Epidemiological Cancer Registry Brandenburg-Berlin were examined separately for adenocarcinoma and squamous cell carcinoma with respect to epidemiology and health care reality.

**Results:**

Between 2000 and 2018 a total of 3207 esophageal cancers were documented in the cancer registry, of which 2182 were squamous cell carcinomas (ESCC), 843 adenocarcinomas (EAC) and 182 various others or missing histology. During the observation period there was a clear dominance of ESCC but with a significant increase in EAC in both sexes. Overall, the rate of new cases was 5 times higher for men than for women. The relative 5‑year survival probability of all esophageal cancers was 17.4% in men and 22.5% in women. Patients with EAC survived significantly longer than those with ESCC. Radiotherapy and chemotherapy, individually or in combination, were mainly used as treatment methods. Surgery was performed on 19% of ESCC and 42% of EAC.

**Conclusion:**

The proportion of ESCC in Brandenburg is still significantly higher than EAC, with a significant increase for the latter, especially in men. Although locally advanced tumors have been significantly more common, modern neoadjuvant concepts have rarely been documented, and although the quality of the surgery is comparable to the international standard, surgery is carried out in relatively few patients.

## Hintergrund

Das Ösophaguskarzinom steht mit weltweit mit 604.100 Neuerkrankungen im Jahr 2020 an Platz 7 der Tumorneuerkrankungen und mit 544.076 pro Jahr an Platz 6 der malignomassoziierten Todesfälle [10]. Die Neuerkrankungsrate wird in Deutschland mit 9,0 pro 100.000 Einwohner für Männer und 2,2 für Frauen (Robert-Koch-Intitut (RKI) 2021) angegeben [12]. Als wesentliche Subtypen liegen vorwiegend proximal gelegene Plattenepithelkarzinome (ESCC) vor und meist distal gelegenen Adenokarzinome (EAC), einschließlich der Tumoren des gastroösophagealen Überganges (AEG Typ 1).

Bei einer globalen Prädominanz an ESCC zeigte sich in verschiedenen westlichen Ländern eine erhebliche Zunahme an EAC [10]. Die histologischen Subtypen sind neben der Lokalisation und dem Tumorstadium entscheidend für die Therapieplanung und die Prognose. Bei fehlender Metastasierung sollte die Möglichkeit einer multimodalen Therapie, einschließlich der Resektion in kurativer Intention, geprüft werden. Beim lokalisierten ESCC ist die definitive kombinierte Radiochemotherapie mit kurativem Ansatz möglich. Für die lokal fortgeschrittenen EAC sind Protokolle mit perioperativer Chemotherapie analog der Behandlung des Magenkarzinoms etabliert [1].

Die palliative Systembehandlung der EAC erfolgt ebenfalls in Anlehnung an die Protokolle der Magenkarzinome [3, 16]. Für die palliative Chemotherapie der ESCC war ein Überlebensvorteil bis zur Einführung der Immuncheckpointinhibitoren nicht gesichert [5, 9, 14, 15].

Seit 1996 werden im Bundesland Brandenburg Daten an das Krebsregister gemeldet. Dies ist die Ausgangsbasis dieser Analyse. Ziel war die Abbildung der lokalen Epidemiologie und der Behandlungsergebnisse in dem Flächenland Brandenburg mit einer vergleichenden Gegenüberstellung von EAC und ESCC.

## Methodik

### Datenerhebung

Die Datenerhebung und Datenauswertung erfolgte über das klinisch-epidemiologische Krebsregister Brandenburg-Berlin (KKRBB) auf Basis einer Auswertungsanfrage der Arbeitsgemeinschaft Gastrointestinale Tumoren Brandenburg.

Erhoben wurden die Daten anhand des ICD-Kodes von als Erstdiagnose gemeldeten Ösophaguskarzinomen (C15) mit Wohnort Brandenburg. Erfasst wurden betroffene Personen mit EAC oder ESCC, andere histologische Entitäten wurden aufgrund der geringen Fallzahl nicht in die Auswertung miteinbezogen.

### Patienten und Zeiträume

Für die Datenauswertung wurden als Neuerkrankung gemeldete Personen mit einem Ösophaguskarzinom der Jahre 2000 bis 2018 herangezogen.

Es wurden rohe Neuerkrankungsraten berechnet, um zeitliche Veränderungen im Auftreten der Krebserkrankungen zu analysieren. Zusätzlich wurden altersstandardisiert Neuerkrankungsraten (nach altem Europastandard, ESR) berechnet. Zur Beschreibung des Überlebens nach einer Krebsdiagnose wurden absolute und relative Überlebensraten für den Zeitraum 2000 bis 2018 berechnet. Die Berechnung der absoluten Überlebensraten erfolgte nach Kaplan-Meier, das relative Überleben wurde mit der Ederer-II-Methode berechnet. Die statistische Beurteilung der Unterschiede erfolgte mittels Log-Rank-Test, des Weiteren erfolgten Analysen mittels Cox-Regression. Zum Vergleich der Verteilung von Kenngrößen bei Gruppen wurde der χ^2^-Test verwendet. Zur Beantwortung der Frage, ob es eine Zunahme von EAC und/oder ESCC im Verlauf der Diagnosejahre 2000 bis 2018 gab, wurden generalisierte additive Modelle (GAM) basierend auf „cubic regression splines“ mit „gamma“ verteilten Residuen verwendet. Für Frauen und Männer wurden jeweils eigene GAM gerechnet; die Histologiegruppen EAC und ESCC sind als Interaktion mit den Diagnosejahren im Modell berücksichtigt. Die Auswertungen erfolgten mit SPSS (Statistics Version 24, SPSS Inc., IBM, Chicago, IL) und RStudio [12].

### Stadien nach UICC

Da sich in den Beobachtungszeiträumen Veränderungen in der Stadieneinteilung durch die Novellierungen der UICC-Klassifikationen (UICC 6–8) insgesamt 4‑mal ergaben, wurden immer die zum Zeitpunkt gültigen UICC-Klassifikationen verwandt. Es erfolgte die Berücksichtigung der unterschiedlichen Einteilung in Bezug auf klinische Stadien.

## Ergebnisse

### Epidemiologischen Daten

Laut Daten des Krebsregisters erkrankten im Zeitraum 2000 bis 2018 *n* = 3207 Männer und Frauen neu an einem Ösophaguskarzinom. Die standardisierte Neuerkrankungsrate pro 100.000 Einwohner (ESR) liegt im Jahr 2018 für Männern bei 8,5 und damit über 5fach höher als bei Frauen mit 1,5. Die Altersverteilung zeigt das bekannte Gewicht der Ersterkrankungen im Bereich des höheren Lebensalters und liegt für Männer im Median bei 65 Jahren und für Frauen im Median bei 70 Jahren.

**Abb. 1:**
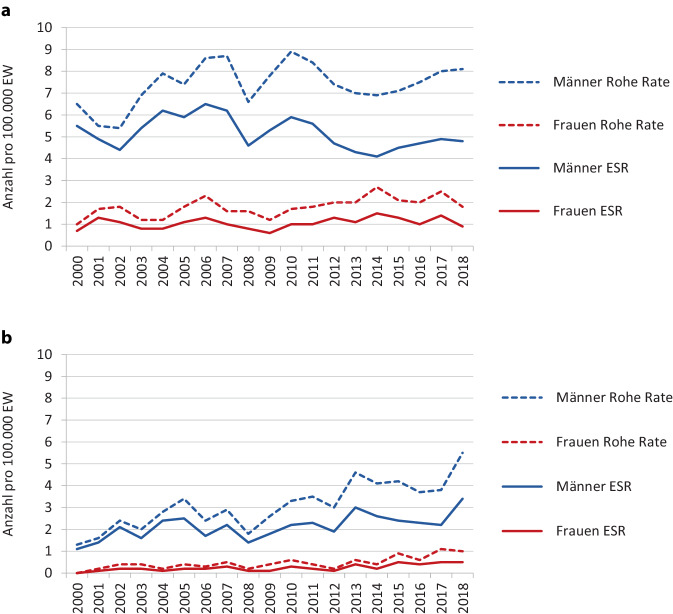
Rohe und standardisierte (ESR) Neuerkrankungsraten für Karzinome des Ösophagus (C15) in Brandenburg. **a** Invasive ESCC, **b** invasive EAC

Im Hinblick auf die histologischen Subtypen dominieren ESCC mit 68 % (*n* = 2182), vor den EAC mit 26 % (*n* = 843). In 182 Fällen (6 %) lagen andere oder fehlende Histologien vor, die bei der weiteren Analyse nicht mitberücksichtigt wurden. Bei Frauen liegt der absolute Anteil an ESCC gegenüber EAC höher (3,8fach) als bei Männern (2,4fach). Für ESCC des Ösophagus konnten weder bei Frauen (F = 1,16, *p* = 0,29) noch bei Männern (F = 1,64, *p* = 0,26) signifikante Einflüsse des Diagnosejahres auf die standardisierten Neuerkrankungsraten (ESR) gefunden werden (Abb. 1a). Bei den EAC lässt sich ein signifikanter positiv linearer Anstieg der standardisierten Neuerkrankungsraten über die Diagnosejahre 2000 bis 2018 für Männer als auch für Frauen verzeichnen (Männer: F = 18,47 *p* < 0,005; Frauen: F = 27,85 *p* < 0,005; Abb. 1b).

### Erkrankungslokalisation

Die Erkrankungslokalisation befindet sich für die EAC vorwiegend mit 64,9 % im unteren Drittel (ICD C15.5) bzw. im abdominellen Anteil (C15.2) des Ösophagus. In 14,5 % der Fälle sind EAC außerhalb der genannten Bereiche in höheren Abschnitten dokumentiert. Die ESCC liegen vorwiegend mit 55 % im Bereich des mittleren und oberen Drittels (C15.4, C15.3) bzw. des thorakalen (C15.1) Anteils des Ösophagus. Hier sind 24,2 % der Fälle im unteren Drittel (C15.5) bzw. im abdominalen Ösophagus dokumentiert (Tab. 1).

**Tab. 1:** ESCC und EAC, Lokalisation nach Histologie

	Plattenepithelkarzinom		Adenokarzinom	
	Anzahl	Prozent	Anzahl	Prozent
C15.2/C15.5 Ösophagus unteres Drittel/abdominaler Ösophagus	529	24,2	547	64,9
C15.0/C15.1/C15.3/C15.4 Ösophagus oberes und mittleres Drittel/zervikaler und thorakaler Ösophagus	1200	55,0	122	14,5
C15.8/C15.9 Ösophagus mehrere Teilereiche/o.n.A.	453	20,8	174	20,6
Gesamt	2182	100	843	100

Wohnort Land Brandenburg, Diagnosejahre 2000 bis 2018, *n* = 3025

### Erkrankungsstadien

**Abb. 2:**
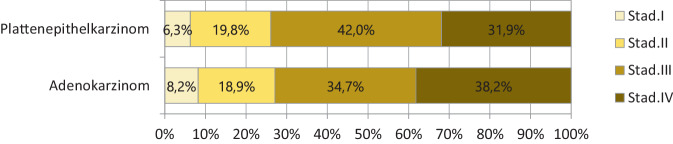
ESCC und EAC des Ösophagus, klinisches Stadium (in jeweils gültiger Auflage), Wohnort Land Brandenburg, Diagnosejahre 2000 bis 2018, *n* = 2275 (ohne Stadium X, k. A., *n* = 750 [24,4 %])

In Bezug auf die Erkrankungsausbreitung bei Erstdiagnose überwiegen die höheren Stadien III und IV ohne signifikante Änderung im Verlauf des Beobachtungszeitraumes. Der Anteil an synchroner Metastasierung (M1) liegt bei EAC bei 25 % und bei ESCC bei 24 %. Bei 22 % (ESCC) bzw. 32 % (EAC) der Fälle wurde bei der primären Datenerhebung kein klinisches Stadium dokumentiert (Abb. 2).

### Überlebensdaten in Abhängigkeit von dem Geschlecht

Für die absoluten Überlebensdaten ergibt sich für das Ösophaguskarzinom insgesamt zwischen den Geschlechtern kein signifikanter Unterschied (*p* = 0,4). Ebenso ergeben sich keine Unterschiede im relativen Überleben. Für alle Ösophaguskarzinome liegt die relative 5‑Jahres-Überlebenswahrscheinlichkeit für Männer bei 17,4 % (CI 13,5–21,3) und für Frauen bei 22,5 % (CI 20,9–24,1).

### Überlebensdaten in Abhängigkeit von dem histologischen Subtyp

Stadienübergreifend zeigt sich in den Diagnosejahren 2000 bis 2018 eine signifikant bessere absolute Überlebenswahrscheinlichkeit für die 843 EAC mit 22 %, im Vergleich zu den 2182 ESCC mit 14 % (*p* < 0,001). Der Unterschied zeigt sich in der Subgruppe der Männer deutlicher (22 % vs. 13 %, *p* < 0,005) als bei Frauen, hier wird kein Signifikanzniveau erreicht (23 % vs. 18 %, *p* = 0,2). Die Verhältnisse zeigen sich vergleichbar im relativen Überleben (Tab. 2).

**Tab. 2:** Absolutes und relatives 5‑Jahres-Überleben in Abhängigkeit von Tumorstadium und Geschlecht

		Plattenepithelkarzinome (%)		Adenokarzinome (%)	
		Abs. 5-J‑Ü	Rel. 5‑J‑Ü	Abs. 5-J‑Ü	Rel. 5‑J‑Ü
UICC	I	28,9	33,6	48,3	59,4
	II	21,4	24,2	23,4	27,7
	II	13,7	15,3	21,5	24,1
	IV	5,4	6,1	4,3	4,8
Geschlecht	Männer	12,7	14,3	21,6	25,0
	Frauen	18,5	20,8	23,5	28,2

### Überlebensdaten in Abhängigkeit von dem Zeitpunkt der Diagnose

Für das ESCC ergibt sich ein absolutes 5‑Jahres-Überleben in den Diagnosejahren 2000 bis 2004 (*n* = 501) von 12 %, 2005 bis 2009 (*n* = 599) von 13 % und 2010 bis 2018 (*n* = 1082) von 15 %. Der Unterschied ist zwischen dem Intervall 2010 bis 2018 und 2000 bis 2004 signifikant (*p* = 0001).

Für das EAC ergibt sich ein absolutes 5‑Jahres-Überleben in den Diagnosejahren 2000 bis 2004 (*n* = 144) von 17 %, 2005 bis 2009 (*n* = 188) 19 % und 2010 bis 2018 (*n* = 511) 24 %. Der Unterschied erreicht zwischen dem Intervall 2010 bis 2018 und 2000 bis 2004 Signifikanz (*p* = 0,04).

### Überlebensdaten in Abhängigkeit von dem klinischen Stadium

**Abb. 3:**
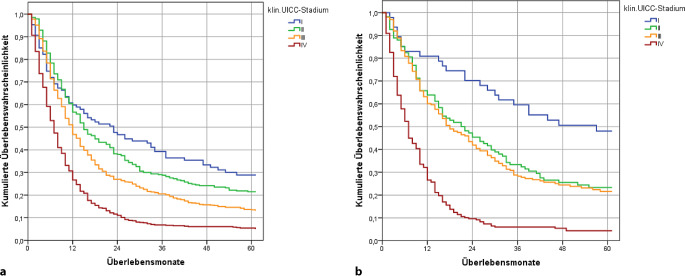
Ösophaguskarzinome Stadium I bis IV, absolutes Überleben nach dokumentiertem klinischem Stadium (in jeweils gültiger Auflage), Wohnort Land Brandenburg, Diagnosejahre 2000 bis 2018. **a** ESCC *n* = 1704, Signifikanzniveau erreicht: Stadium II vs. III, Stadium III vs. IV. **b** EAC *n* = 571, Signifikanzniveau erreicht: Stadium I vs. II, Stadium III vs. IV

Das 5‑Jahres-Überleben zeigt in beiden Entitäten jeweils eine Abhängigkeit von dem klinischen Tumorstadium nach UICC (*p* < 0,001) und von dem Geschlecht (Tab. 2 und Abb. 3).

### Ösophaguskarzinome nach Primärtherapie

#### Primärtherapie nach klinischem Stadium bei ESCC

**Abb. 4:**
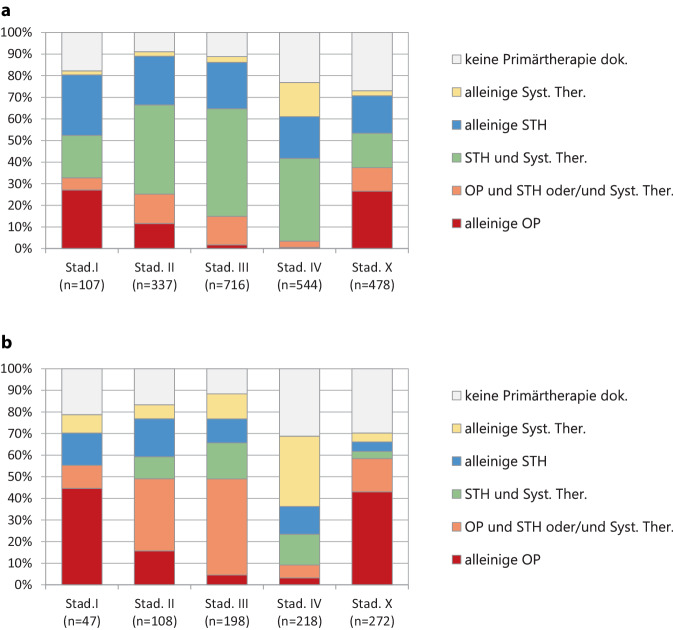
Primärtherapie nach klinischem Stadium, Karzinome des Ösophagus, Wohnort Land Brandenburg, Diagnosejahre 2000 bis 2018. **a** ESCC *n* = 2182, **b** EAC *n* = 843

Im Stadium I dominiert bei kleiner dokumentierter Fallzahl (*n* = 88) die Strahlen- und die kombinierte Radiochemotherapie vor der alleinigen Operation (Tumorresektion). Der Anteil der Fälle, die im Stadium I nicht operiert und nur einer alleinigen Strahlen- oder Systemtherapie zugeführt wurden, liegt bei 29,9 % mit einem höheren medianen Lebensalter (71 vs. 62 Jahre). Die alleinige Operation kommt bei vergleichender Betrachtung aller UICC-Stadien im Stadium I am häufigsten vor (Abb. 4a). Der Anteil alleiniger Radiatio mit systemischer Therapie gegenüber einer Operation mit Radiatio und systemischer Therapie ist im Stadium II höher als im Stadium III. Im Stadium II wurden 24,6 % nur einer alleinigen Strahlen- oder Systemtherapie zugeführt bei einem höheren medianen Lebensalter (70 vs. 64 Jahre). In beiden Therapiegruppen überwiegen ECOG 0 und 1, sie dominieren jedoch bei der Therapiegruppe Operation mit Radiatio und systemischer Therapie deutlicher (Tab. 3). Ohne Berücksichtigung des Anteils, bei dem keine Therapie gemeldet wurde, wurde insgesamt ein operatives Konzept im Stadium I bei *n* = 35/88 (40 %) im Stadium II bei *n* = 85/307(28 %) und im Stadium III bei *n* = 107/636 (17 %) der Fälle durchgeführt. Auch im Stadium IV dominieren die strahlentherapeutischen Konzepte vor der Chemotherapie.

**Tab. 3:** ECOG nach Therapiegruppen, klinisches Stadium II oder III mit Radio- und Chemotherapie (*n* = 252) oder Operation, Radio- und Chemotherapie (*n* = 79), ESCC des Ösophagus, ohne fehlende ECOG-Werte, Wohnort Land Brandenburg, Diagnosejahre 2000 bis 2018, *n* = 331, χ^2^-Test *p* < 0,001

	ECOG 0 (%)	ECOG 1 (%)	ECOG 2 (%)	ECOG 3 (%)
STH und syst. Ther	19,8	47,6	26,6	6,0
OP, STH und Syst. Ther	39,2	46,8	13,9	0

#### Primärtherapie nach klinischem Stadium bei EAC

Bei kleiner Fallzahl ist im Vergleich zum ESCC die alleinige Operation im Stadium I das häufigste Vorgehen. Der Anteil der Fälle, die im Stadium I nicht operiert und nur eine alleinige Strahlen- oder Systemtherapie erhielten, liegt bei 23,4 % mit einem höheren medianen Lebensalter (69,5 vs. 77,8 Jahre). Insgesamt wurde (ohne Berücksichtigung nicht gemeldeter Therapien) ein operatives Konzept im Stadium I bei *n* = 31/54 (57 %), im Stadium II bei *n* = 56/112 (50 %) und im Stadium III bei *n* = 92/194 (47 %) der Patienten durchgeführt. Damit wurde prozentual wesentlich häufiger operiert als beim ESCC. 24 % erhielten im Stadium II eine alleinige Strahlen- oder Systemtherapie bei höherem Lebensalter (Median 77 vs. 64 Jahre). Im Stadium II und III ist ein multimodales Therapiekonzept mit Operation, Radiatio und systemischer Therapie die führende Therapie. Erst im Stadium IV überwiegt die alleinige systemische Therapie (Abb. 4b).

#### Überlebensdaten in Abhängigkeit von Primärtherapie bei ESCC

**Abb. 5:**
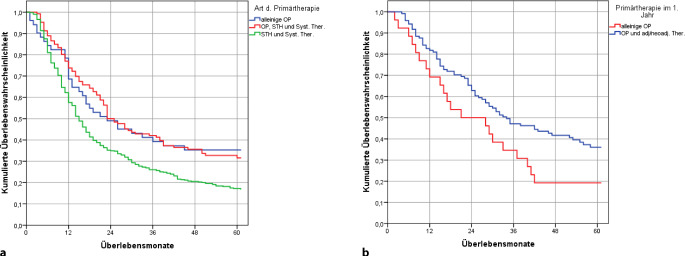
Absolutes OAS nach Primärtherapie, Karzinome des Ösophagus Wohnort Land Brandenburg, Diagnosejahre 2000 bis 2018. **a** ESCC, *n* = 621 klinisches Stadium II oder III mit Radio- und Chemotherapie (*n* = 495) oder Operation, Radio- und Chemotherapie (*n* = 126) oder alleiniger Operation (*n* = 51), **b** EAC, *n* = 147 klinisches Stadium II oder III mit alleiniger Operation (*n* = 26) und Operation + adjuvante/neoadjuvante Therapie (*n* = 121)

Das absolute Überleben ist in der Gruppe der Patienten nach alleiniger Operation oder nach multimodaler Therapie und Operation höher als in der Gruppe nach alleiniger Radiochemotherapie (Abb. 5a).

#### Überlebensdaten in Abhängigkeit von Primärtherapie bei EAC

Im klinischen Stadium II und III ist das absolute Überleben nach einem multimodalen Konzept besser als nach alleiniger Operation (*p* = 0,021), einschränkend ist dabei der geringe Anteil der Fälle (*n* = 26), die über den gesamten Zeitraum rein operativ versorgt wurden (Abb. 5b).

#### Daten zur Operation

**Abb. 6:**
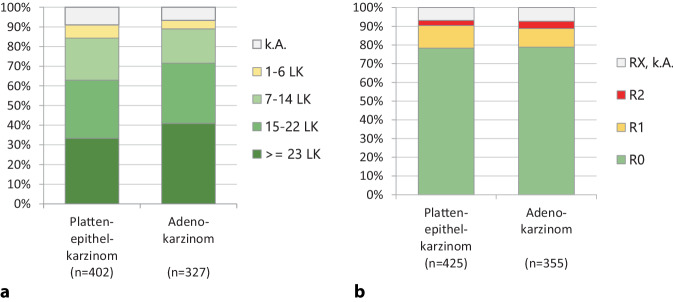
Operationsergebnisse Karzinome des Ösophagus, Wohnort Land Brandenburg, Diagnosejahre 2009 bis 2018. **a** Anzahl der entfernten Lymphknoten: ESCC (*n* = 402) und EAC (*n* = 327), χ^2^-Test *p* = 0,175, **b** globale R‑Klassifikation: ESCC (*n* = 425) und EAC (*n* = 355), χ^2^-Test *p* = 0,658

Insgesamt wurden nur wenige Patienten einer tumorresezierenden Operation unterzogen. So wurden nur 20 % der 2182 ESCC reseziert und 42 % der 843 EAC. Der Anteil einer endoskopischen Versorgung lag im Stadium I bei 31 %. Es wurde eine Mindestanzahl von 7 Lymphknoten (LK) beim ESCC in 92,6 % und beim EAC in 95,4 % reseziert (ohne Rx, k. A). Mehr als 15 LK wurden beim ESCC in 69,1 % und beim EAC in 76,7 % und mehr als 23 LK beim ESCC in 36 % und beim EAC in 43,9 % entnommen (Abb. 6a). Als Resektionsergebnis konnte in 78 % bei den ESCC eine R0-Resektion erzielt werden. Bei den EAC wurden mit 79 % ähnliche Ergebnisse erzielt (Abb. 6b). R0-resezierte Patienten (*n* = 583) überlebten mit einer 5‑Jahres-Überlebensrate von 41 % signifikant besser als nicht-R0-resezierte (R1, Rx, k. A. *n* = 130) mit einer 5‑Jahres-Überlebensrate von 18 %.

## Diskussion

Die für die Entität typische Geschlechterverteilung bei den Erkrankungsraten zeigt sich innerhalb der letzten 20 Jahre stabil. In der Betrachtung der histologischen Subgruppen liegt für die Region eine klare Prädominanz für das ESCC vor. In einigen hoch entwickelnden Ländern in Nordeuropa, Nordamerika, Australien und Kanada dominieren EAC als histologischer Subtyp [10]. In den USA kam es zu einem deutlichen Anstieg der EAC insbesondere bei Männern im zeitlichen Verlauf bis 2007 und einem zeitgleichen Rückgang der ESCC [2]. Daten des Zentrums für Krebsregisterdaten zeigen für Deutschland eine vergleichbare Entwicklung mit einer beginnenden Prädominanz an EAC vor den ESCC für Männer (2015–2016; [8]). Im Gegensatz dazu, lässt sich in Brandenburg für die EAC zwar ein ähnlicher Trend nachvollziehen, jedoch in einem deutlich geringeren Ausmaß und bei einer gleichbleibenden Neuerkrankungsrate für ESCC.

Es zeigt sich an dem Gesamtkollektiv ein günstigeres Überleben für Frauen als für Männer. Das relative 5‑Jahres-Überleben liegt dabei im Abgleich mit dem amerikanischen Kollektiv der SEER-Database (2000–2019) mit 22,5 % für Frauen etwas höher (SEER 20,0 %) und mit 17,4 % für Männer etwas niedriger (SEER 18,9 %; [13]). Hierbei ist zu berücksichtigen, dass der Anteil der EAC in den amerikanischen Daten bei Männern über dem der ESCC liegt. Das 5‑Jahres-Überleben ist für beide histologischen Hauptentitäten von den Erkrankungsstadien abhängig. Bei beiden Geschlechtern ist das Überleben der EAC günstiger als in den SEER-Daten (Männer: 25,0 % vs. 21,1 %, Frauen: 28,2 % vs. 20,8 %), während sich die Überlebensdaten bei den ESCC nicht unterscheiden. Vergleichbar mit den SEER-Daten ist das Überleben in der Gruppe der Männer besser für den EAC-Subtyp. Hingegen zeigt sich bei Frauen das bessere Überleben der EAC gegenüber den ESCC diskrepant zu den SEER-Daten (hier kein Unterschied; [13]). Das Überleben in den metastasierten Stadien ist für beide Entitäten vergleichbar schlecht (SEER EAC 5,1 %, ESCC 5,9 %) und liegt in den lokalisierten und regionalen Stadien in einem ähnlichen Bereich [13]. Das geschlechterabhängige Überleben in den Subentitäten könnte unter anderem in regional unterschiedlichen Erkrankungsauslösern und Risikofaktoren zwischen Frauen und Männern liegen, insbesondere bei den ESCC.

Im Verlauf der betrachteten Zeitabschnitte (2000–2004, 2010–2018) zeigt sich zwar für beide Entitäten bei relativ konstant gebliebener Stadienverteilung eine Verbesserung des Gesamtüberlebens, wir können anhand der vorliegenden Daten die Ursachen hierfür nicht benennen. In den USA zeigt sich ein vergleichbarer Trend (2000–2020).

Für die ESCC ist der Anteil an multimodalen und an chirurgischen Therapiekonzepten gering. Der Anteil an Fällen, die im Stadium I und II keiner definitiven Therapie zugeführt wurden, ist auffallend hoch. Zur Orientierung lag der Anteil der Fälle (Ösophagus gesamt) mit T1N0, die im Auditjahr 2020 der Deutschen Krebsgesellschaft (DKG) nicht operiert wurden, bei 6,43 % und mit T2,T3N0 bei 8,81 % [7]. In beiden Subentitäten findet sich für die nicht definitiv behandelten als mögliches Argument ein höheres medianes Lebensalter, signifikante Unterschiede im ECOG-Staus ließen sich bei kleiner Fallzahl nicht abgrenzen.

Die R0-Resektionsrate ist zwar im Laufe der Jahre von 57,6 % (2000) auf 89,1 % (2018) gestiegen, jedoch sind die durchschnittlichen R0-Resektionsraten im genannten Zeitraum von 78 % für ESCC und 79 % für EAC im Vergleich zum Jahresbericht der DKG von 2021 mit 95 % zu niedrig. Laut Leitlinien zur Behandlung des Ösophaguskarzinoms stellt die 2‑Feld-Lymphadenektomie (LA) den Standard dar, wobei sich das Ausmaß der LA nach der Lokalisation des Primärtumors richtet. Konkrete Zahlen werden in der Leitlinie nicht gefordert, aber 7 LK sind zunächst für die TNM-Klassifikation erforderlich. Zwar wurden diese beim ESCC in 92,6 % und beim EAC in 95,4 % erreicht, in der Realität sind jedoch eher 15 LK prognostisch relevant. In einer Multicenterstudie zeigte sich sogar, dass die Zahl der entnommenen Lymphknoten ab 23 ein unabhängiger Prädiktor für das Überleben sein kann [4, 11]. Mehr als 15 LK wurden beim ESCC nur in 69,1 % und beim EAC in 76,7 % und mehr als 23 LK beim ESCC gar nur in 36 % und beim EAC in 43,9 % entnommen. Dies ist nach dem heutigen Standard als zu wenig anzusehen.

Da die DKG-Zertifizierung für das Modul Speiseröhre erst ab 2018 möglich wurde, ist eine Gegenüberstellung von Daten zertifizierter und nichtzertifizierter Zentren innerhalb des untersuchten Auswertungszeitraumes nicht aussagekräftig.

In den Krebsregisterdaten sind Begleiterkrankungen und Sozialstatus nicht enthalten, die älteren Daten weisen einen hohen Anteil an fehlendem ECOG auf, sodass auch multivariate Analysen eine begrenzte Aussagekraft haben. Insbesondere für die EAC zeigt sich, dass ein an Stadien adaptiertes multimodales Konzept nicht konsequent durchgeführt wurde. Auch hier lassen sich mögliche Gründe wie Begleiterkrankungen oder regionale Versorgungsdefizite innerhalb von Brandenburg nicht abgrenzen.

## Schlussfolgerung

Zusammenfassend werden anhand der vorliegenden Daten die biologischen Unterschiede der Ösophaguskarzinome deutlich, die eine getrennte Betrachtungsweise für EAC und ESCC verlangen. Die Überlebensdaten weisen auf eine vergleichbare Versorgung wie beispielsweise in den USA hin. Es besteht Optimierungsbedarf sowohl in der operativen als auch in der perioperativen, stadiengerechten Therapie, auch wenn im Laufe der Beobachtung hier durchaus Verbesserungen zu beobachten sind. Es ist zu erwähnen, dass im Zeitraum 2000 bis 2018 die Daten insbesondere in den früheren Diagnosejahren nicht immer vollständig vorhanden waren und die Fallzahlen für die Untersuchung spezifischerer Fragstellungen zur Therapie gering sind. Für eine künftige Betrachtung wäre die Zusammenführung von Daten mehrerer Landeskrebsregister eine Option. Mit der neuen Version des onkologischen Basisdatensatzes werden auch molekulargenetische Marker erhoben werden, was eine differenziertere Betrachtung von Therapiestrategien ermöglichen wird [6].
